# Metal–Organic Polyhedra as Building Blocks for Porous Extended Networks

**DOI:** 10.1002/advs.202104753

**Published:** 2022-02-04

**Authors:** Akim Khobotov‐Bakishev, Laura Hernández‐López, Cornelia von Baeckmann, Jorge Albalad, Arnau Carné‐Sánchez, Daniel Maspoch

**Affiliations:** ^1^ Catalan Institute of Nanoscience and Nanotechnology (ICN2) CSIC and The Barcelona Institute of Science and Technology Campus UAB, Bellaterra Barcelona 08193 Spain; ^2^ Centre for Advanced Nanomaterials and Department of Chemistry The University of Adelaide North Terrace Adelaide South Australia 5000 Australia; ^3^ Catalan Institution for Research and Advanced Studies (ICREA) Pg. Lluís Companys 23 Barcelona 08010 Spain

**Keywords:** gels, membranes, metal–organic frameworks, metal–organic polyhedra, porous networks, reticular chemistry

## Abstract

Metal–organic polyhedra (MOPs) are a subclass of coordination cages that can adsorb and host species in solution and are permanently porous in solid‐state. These characteristics, together with the recent development of their orthogonal surface chemistry and the assembly of more stable cages, have awakened the latent potential of MOPs to be used as building blocks for the synthesis of extended porous networks. This review article focuses on exploring the key developments that make the extension of MOPs possible, highlighting the most remarkable examples of MOP‐based soft materials and crystalline extended frameworks. Finally, the article ventures to offer future perspectives on the exploitation of MOPs in fields that still remain ripe toward the use of such unorthodox molecular porous platforms.

## Graphical abstract

Recent advances in the robustness, solubility and reactivity of metal–organic polyhedra (MOPs) have enabled their use as porous building blocks for the synthesis of extended, hierarchical porous networks. The key developments making the extension of MOPs feasible and the type and properties of the obtained porous networks are covered in this review.

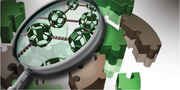

## 1 Introduction

The development of new materials with benchmark physical properties is imperative for the chemical industry. To this end, researchers must gain fundamental insights into self‐assembly and create predictive design principles for structure‐function relationships. Advances in both areas have recently been reported for porous materials, for which establishing the relationship between physical properties and pore chemistry is crucial.^[^ ^1^, ^2^, ^3^ ^]^ Porous materials have garnered attention from both industry and academia due to their outstanding structural diversity and versatility,^[^ ^4^, ^5^, ^6^, ^7^, ^8^, ^9^ ^]^ which makes them attractive candidates for applications such as gas sorption/separation,^[^ ^10^, ^11^, ^12^, ^13^, ^14^ ^]^ heterogeneous catalysis^[^ ^15^, ^16^, ^17^, ^18^ ^]^ and sensing.^[^ ^19^, ^20^ ^]^ The chemistry of porous materials has matured significantly over the past decade, thanks to the introduction of supramolecular chemistry as a synthetic tool for introducing pre‐organized subunits within extended materials.^[^ ^21^, ^22^, ^23^ ^]^ Thus, researchers have developed an extensive library of synthetic (and post‐synthetic) strategies that, unlike standard one‐step syntheses, aim to control the assembly of elaborated architectures with well‐defined chemistry in a stepwise fashion.^[^ ^24^, ^25^, ^26^ ^]^ Although these multistep approaches are generally tedious, they generate materials with unparalleled molecular precision at one or more porous domains, owing to the greater levels of pre‐organization during self‐assembly than those seen in one‐step syntheses.

The judicious assembly of pre‐synthesized molecular building blocks into porous architectures has enabled an unprecedented degree of control over the structure and composition of porous materials, as exemplified by the success of reticular chemistry to engineer metal–organic frameworks (MOFs) with unprecedented features such as hierarchical porosity.^[^ ^27^, ^28^ ^]^ Hierarchically porous materials possess a complex structure with multiple micro‐, meso‐ and macroporous domains that exhibit highly distinct chemistries and functionalization.^[^ ^29^, ^30^ ^]^ These materials have attracted interest for their unique performance during controlled transport of substrates throughout their frameworks, which is heavily influenced by the degree of order in their 3D structures.^[^ ^31^, ^32^ ^]^ Among the most promising—albeit underexplored—routes to generate multiple well‐defined porous domains is the stepwise assembly of intrinsically porous building blocks (i.e., the spatial polymerization of preformed porous cages).^[^ ^33^ ^]^ This methodology arguably offers the highest level of hierarchical control, in which the pore's inner chemistries (windows, diameter, polarity) and outer chemistries (functionalization, directionality) are strategically pre‐established to confer the resultant material with at least two well‐defined chemical domains.^[^ ^34^ ^]^ Furthermore, the better defined the reactivity of these porous building blocks, the greater the precision at which they can be interconnected for specific applications without disturbing their core functionalities.^[^ ^35^ ^]^

Metal–organic polyhedra (MOPs) have become one of the most attractive classes of building blocks for the growth of hierarchically controlled, metal–organic, porous materials.^[^ ^36^, ^37^ ^]^ MOPs are a subclass of coordination cages, which present the distinctive feature of being permanently porous in solid state.^[^ ^38^ ^]^ The molecular nature of MOPs and their hybrid metal–organic surface endow these materials with a rich orthogonal surface reactivity that is accessible both in solution and in the solid‐state. Furthermore, the number and location of the different functional groups on the surface of the MOP can be precisely known. This enables very fine control over their processability, stability, and chemical reactivity while maintaining their porosity.^[^ ^39^ ^]^ The unique combination of intrinsic porosity with a well‐defined surface reactivity is what makes MOPs ideal candidates to be used as porous monomeric units for the assembly of extended porous networks.

The use of MOPs as building blocks for porous networks presents several advantages such as the possibility to confer extended materials with permanent porosity regardless of the final degree of order of these materials, since both the functionalization and integrity of the cage—particularly for those with strong metal–ligand coordination bonds—are thoroughly maintained during the polymerization step.^[^ ^40^ ^]^ This in turn paves the way for the controlled embedding of porosity into soft matter such as gels or self‐assembled monolayers. Alternatively, the possibility to precisely locate reactive sites on the polyhedral surface of MOPs with accurate knowledge of their number, location, and orientation enables the use of MOPs as pre‐synthesized supermolecular building blocks that can be assembled into highly connected crystalline nets via reticular chemistry.^[^ ^41^ ^]^ This approach was recently employed to target such nets, which could not be readily accessed through conventional syntheses.^[^ ^42^ ^]^

Theoretically, the hierarchical assembly of MOPs should offer tremendous advantages over more conventional one‐pot syntheses in reticular crystalline frameworks as well as in soft/amorphous materials. However, the use of MOPs as monomers in subsequent self‐assembly/polymerization reactions has been long hindered by three deficiencies: lack of solubility in desired solvents, poor stability, and limited reactivity.^[^ ^43^ ^]^ Fortunately, all three issues are being addressed by researchers, who have engineered a new generation of MOPs that combine high structural stability with surfaces amenable to functionalization.^[^ ^44^, ^45^, ^46^, ^47^ ^]^ Moreover, the synthetic strategies to assemble such MOPs have been flourishing: indeed, MOPs assembled through coordination, supramolecular, and/or covalent interactions have recently been reported. These MOPs are being harnessed to synthesize an exciting variety of porous materials that are well‐poised to advance the field of porous networks. In this context, this review article is poised to guide the lector through the main factors that contributed to bracing MOP toward becoming excellent reticular building blocks for the assembly of complex porous extended architectures, from soft matter to crystalline frameworks.

## 2 The Building Block: Metal–Organic Polyhedra

**Figure 1:**
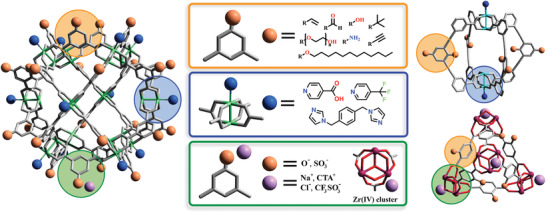
Schematic representation of the reactive sites on the surface of MOPs: functional pendant groups on the organic ligand (highlighted in orange), coordinated ligands on the open metal sites (highlighted in blue), and counter‐ions in charged MOPs (highlighted in green).

As a subclass of coordination cages, MOPs employ directional metal–ligand coordination bonds that can be exploited for the design and synthesis of discrete molecular architectures that possess internal cavities. However, MOPs differ from other coordination cages in that their cavities are preserved upon activation or desolvation, making them permanently porous in the solid‐state.^[^ ^48^, ^49^ ^]^ This characteristic stems from the strong metal–ligand (typically, metal–carboxylate) coordination bonds that sustain their structure. Therefore, MOPs can be classified as reticular materials, owing to the presence of strong and directional bonds that lead to permanently porous materials. Accordingly, one can subject MOPs to reticular chemistry, such as the secondary building unit (SBU) approach, to generate discrete polyhedral architectures, as exemplified by the assembly of M(II) paddlewheel clusters with bent dicarboxylate ligands. This paddlewheel can be conceptualized as a 4‐connected (4‐c) square planar unit. Accordingly, the assembly of M(II) paddlewheels with bent ligands can be anticipated by considering the angle between the two carboxylate groups. The most representative MOPs obtained with these building blocks are the lantern‐type (general formula M_2_L_4_; 0 ° angle between carboxylic groups), the octahedral‐type (general formula M_12_L_12_; 90 ° angle between carboxylic groups), and the cuboctahedral‐type (general formula M_24_L_24_; 120 ° angle between carboxylic groups).^[^ ^37^ ^]^ This approach has been progressively expanded to other metallic clusters of higher nuclearity, including iron‐ or zirconium‐capped trimers, and calixarene‐capped tetranuclear clusters.^[^ ^46^, ^50^, ^51^ ^]^ The defined position of each building block in a final MOP structure enables the use of reticular chemistry to functionalize, with atomic precision, the MOP's internal cavity and external surface. For example, the 5‐position of the benzene ring of the 1,3‐benzenedicarboxylic acid (BDC) has been used to functionalize the surface of cuboctahedral MOPs with up to 24 functional groups of diverse nature (**Figure** **1**, orange).^[^ ^52^ ^]^ In the case of reactive moieties, the scope of the MOP surface functionality can be further expanded through covalent post‐synthetic modification reactions. Such control over the surface chemistry of MOPs enables tuning of their physicochemical properties such as solubility, reactivity, and chemical stability, making MOPs a modular porous unit with unique features for hierarchical self‐assembly.

## 3 MOPs as Porous Building Blocks for Hierarchical Self‐Assembly

The use of MOPs as molecular porous building blocks for bottom‐up assembly of extended materials rests on three pillars: stoichiometric reactivity, stability, and solubility. Each of these three requirements ensures that the final extended material is obtained through the linkage of pre‐designed MOPs. Moreover, the hierarchical assembly of soluble MOPs with well‐defined reactive sites enables a better reaction design and rationalization of the structure/properties of the finally accessed porous network. The aforementioned pre‐requisites can be inherently present in the as‐synthetized MOPs thanks to the properties that their molecular constituents confer to them, or can be introduced a *posteriori* through post‐synthetic modification. In the following section, we describe the different routes explored to develop robust, soluble, and reactive MOPs that can subsequently be used as building blocks in the synthesis of hierarchical porous networks.

### 3.1 The Surface Reactivity of MOPs

MOPs inherit distinctive elements from both MOFs and coordination cages, such as open metals sites and organic pendant groups. Therefore, MOPs are potentially reactive through their exposed organic functional groups and/or their metallic nodes. Remarkably, both surface‐reactive organic groups and metal sites generally exhibit orthogonal reactivity, meaning that they can be targeted independently or in tandem. Surface organic groups have been employed to endow MOPs with both covalent and/or coordination reactivities. For example, covalent reactivity has been introduced to MOPs by derivatizing their surfaces with amines, alcohols, alkyne, alkene, aldehyde or dithiobenzoate groups,^[^ ^53^ ^]^ among other moieties, thereby enabling subsequent chemistry with the resultant MOPs, including condensations, click chemistry, olefin metathesis or polymerizations (Figure 1, orange). Similarly, MOPs have been functionalized with organic functional groups with coordination capabilities such as amines, hydroxyls, and carboxylates. Thus, further coordination towards additional metal ions allows the use of coordination chemistry in MOP surfaces. In both cases, reactive groups have typically been introduced onto the surface of the MOP through direct synthesis by selecting ligands bearing such reactive moieties as pendant groups.^[^ ^52^ ^]^ However, in some cases, such as with free amines and carboxylic acids, functionalized MOPs could not be directly synthesized, thus highlighting a demand for alternative approaches based on using protecting groups.^[^ ^54^ ^]^ Interestingly, MOPs functionalized with coordinating groups can undergo supramolecular polymerization via self‐condensation reactions or upon coordination with additional metal ions.^[^ ^42^, ^55^ ^]^

The reactivity of open metal sites in MOPs has been exploited mainly for M(II) paddlewheel‐based MOPs (Figure 1, blue). Paddlewheel clusters have a well‐established reactivity at their axial sites, which can undergo ligand‐exchange reactions with N‐based ligands such as pyridines, amines, or imidazoles without compromising the equatorial M(II)‐carboxylate coordination.^[^ ^56^, ^57^ ^]^ Accordingly, these open metal sites can be used as anchoring points to functionalize the surface of paddlewheel‐based MOPs or to link them with (monodentate or bidentate) N‐based ligands. This chemistry has been explored mainly for Cu(II)‐based MOPs (named *Cu‐MOPs*), although it has recently been expanded to Rh(II)‐based MOPs (named *Rh‐MOPs*).^[^ ^38^ ^]^ In addition to covalent and coordination chemistries, MOPs can also participate in two other supramolecular interactions: electrostatic interactions and H‐bonding. Electrostatic charge has been introduced into MOPs by using intrinsically charged clusters, as has been done with a zirconocene cluster,^[^ ^58^ ^]^ and by using charged organic functionalities such as sulfonate groups (Figure 1, green).^[^ ^59^ ^]^ Charged MOPs can then undergo metathesis for tuning of surface chemistry or induction of self‐assembly. Similarly, the possibility to functionalize the surface of MOPs with H‐bond acceptor/donor groups opens the door to guiding their assembly through complementary H‐bonding interactions.

MOPs can be linked through various supramolecular and covalent interactions provided that their structural integrity is not compromised by such polymerization reactions. The design of such supramolecular or covalent polymerization reactions benefits from precise knowledge of the orientation, number, and location of the reactive sites on the polyhedral surface. Therefore, the geometrical features of the reactive sites, and the type of linkage chemistry, can be strategically combined to dictate the structural features of the resultant network at the molecular scale (connectivity and periodicity), the mesoscale (hierarchical porosity), and the macroscale (shape and mechanical properties). We address these topics in the following sections.

### 3.2 Stability

**Figure 2:**
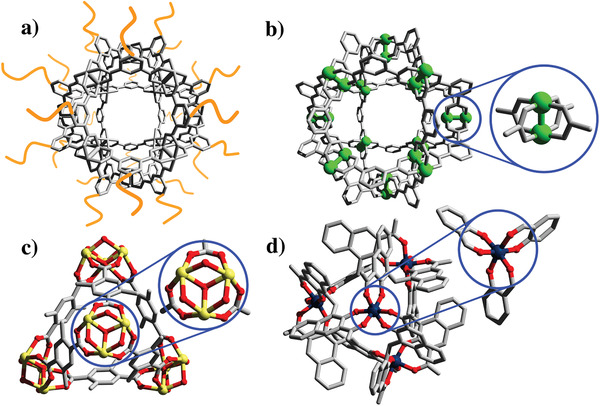
Main strategies to increase MOP stability. a) Shielding of the metallic nodes with hydrophobic or hydrophilic polymeric chains. b) Use of metal ions with strong intermetallic bonds [i.e., Mo(II), Rh(II), Ru(II) or Cr(II)]. c) Increasing metal–ligand coordination bond using strongly coordinating hard‐metal ions in combination with hard bases (O‐donor ligands) (i.e., zirconium clusters). d) Introduction of chelating groups.

In this section, we focus on the stability of MOPs in solution. Here, we define the stability of a MOP as the conservation of a distinguishable 0D entity throughout the entire “cage‐to‐network” reaction (i.e., without any disassembly or re‐assembly of the MOP constituents). The synthesis of MOPs relies on the reversibility of the coordination bonds that sustain their structure.^[^ ^60^, ^61^, ^62^ ^]^ However, this reversibility is also a source of concern when assessing their stability, because they can dissociate in the presence of competing coordinating molecules such as water (i.e., hydrolysis) or additional ligands (i.e., ligand exchange).^[^ ^63^, ^64^ ^]^ Therefore, strategies to augment the robustness of MOPs in solution focus on either shielding the metallic node, using metal ions with strong intermetallic bonds, increasing the coordination strength of the metal–ligand coordination bond, or using chelating groups (**Figure** **2**).

The most frequently used metallic node in MOP chemistry is the Cu(II) paddlewheel. However, both the chemical stability and hydrolytic stability of Cu‐MOPs are compromised by the lability of the Cu‐carboxylate bond, especially its tendency to undergo both hydrolysis and ligand exchange.^[^ ^59^ ^]^ Consequently, the scope of reactivity of Cu‐MOPs toward coordination and covalent polymerization reactions may be limited. Among the most successful approaches to increase the stability of Cu‐MOPs is to preclude the interaction of external agents with the labile nodes, whereby the high surface density of functional groups on the MOP is exploited to shield the vulnerable Cu‐carboxylate bonds from hydrolysis or ligand exchange. To this end, a surrounding organic shell is attached to the rigid organic backbone of the MOP (Figure 2a). Depending on the shell used, the shielding can be either hydrophilic or hydrophobic.

The protective effect of densely grafted hydrophilic polymers was first suggested by Zhou et al., who covalently attached polyethylene glycol chains (exhibiting 5 kDa as molecular weight, *PEG5k*) to a cuboctahedral Cu‐MOP (named *pi‐CuMOP*; also known as Cu(pi)) formulated as [Cu_2_(pi)_2_]_12_ (in which *pi* refers to the ligand 5‐(prop‐2‐ynoxybenzene)‐1,3‐dicarboxylic acid), via a copper(I)‐catalyzed, azide‐alkyne cycloaddition.^[^ ^65^ ^]^ The resultant structure, denoted as *PEG5k‐CuMOP*, demonstrated superior stability to the starting material, as demonstrated in a 24 h aqueous dialysis experiment. The authors attributed this improvement to intermolecular aggregation between soluble entities, which prevented the water molecules from interacting with the Cu(II) paddlewheel.^[^ ^65^ ^]^ The same approach was later used by Yin et al. using poly(*N*‐isopropylacrylamide) (*PNiPAM*) as a polymeric chain instead of PEG.^[^ ^66^ ^]^

Hydrophobic shielding also has been used to modulate the permeability of water molecules into a MOP structure.^[^ ^67^ ^]^ For example, Ghosh et al. demonstrated that attaching hydrophobic moieties to the exposed surface of a cuboctahedral Cu‐MOP enhanced its hydrolytic stability.^[^ ^67^ ^]^ The authors synthesized neutral cages based on 12 Cu(II) paddlewheels and 24 ligands, with a naphthalene diimide core and different pendant amino acids (alanine, valine, isoleucine, and phenylalanine) that each provided a different degree of hydrophobicity to the MOP. They then tested the stability of the resultant MOPs heterogeneously in aqueous media under alkaline, acidic, oxidizing, reducing, and buffered conditions. Owing to the blocking of the paddlewheel units, the MOPs bearing the most hydrophobic groups fully retained their structures in solid state, even after several weeks under the test conditions. However, the potential of this approach for solubilized MOPs, in which the diffusion of water molecules toward the MOP core is less hindered, is unknown.^[^ ^67^ ^]^

The aforementioned examples demonstrate the potential of shielding strategies to protect Cu(II) paddlewheel units in MOPs and consequently, to enhance the stability of MOPs toward hydrolysis in more aggressive media (i.e., alkaline, acidic, etc.). However, the effects of organic shielding on the reactivity of MOPs in polymerization reactions, particularly those based on the reactivity of open metal sites, remain unclear. Contrariwise, covalent polymerization reactions using polymeric/macromolecular ligands can be used to link MOPs together, maintaining the hydrophobic/hydrophilic shielding of open metal sites.

Given the widespread use of the 4‐c paddlewheel cluster in the synthesis of MOPs, researchers have sought to generate an equivalent SBU made of metal ions with a stronger metal–metal bond strength, as a way to maximize overall robustness, including resistance to hydrolysis (Figure 2b). For instance, paddlewheel units containing intermetallic bonds such as those based on Mo(II), Ru(II), Cr(II), or Rh(II), have been described to afford more robust MOPs in the solid‐state, as reflected by their large surface areas (order of magnitude: 1000 m^2^ g^–1^).^[^ ^44^, ^45^, ^49^, ^68^, ^69^ ^]^ In particular, Rh‐MOPs are highly stable in solution under various conditions, including in the presence of coordinating ligands, at high temperature, and in aqueous solutions at extreme pH levels.^[^ ^38^, ^70^, ^71^ ^]^ The outstanding stability of Rh‐MOPs stems from the cooperative strength of the Rh‐carboxylate coordination bond and the Rh‐Rh intermetallic bond, which lock the equatorial coordination sites at room temperature, thereby preventing the cage from undergoing ligand‐exchange reactions.

Another strategy to improve the stability of coordination compounds is to increase the strength of the metal–ligand coordination bond.^[^ ^72^ ^]^ The Zr‐O coordination bond has a high dissociation energy (≈776 kJ·mol^–1^), making it exceptionally resilient to hydrolysis in a wide pH range (pH 1‐10).^[^ ^73^ ^]^ Furthermore, metallic centers with high coordination numbers can generate high nuclearity clusters, thereby yielding remarkably stable M‐L nodes. For this, Zr(IV) clusters are regularly used to provide chemical stability—especially hydrolytic stability—to metal–organic materials (typically: MOFs).^[^ ^74^ ^]^ Recently, Zr(IV)‐carboxylate chemistry was implemented to synthesize Zr(IV)‐based MOPs (named *Zr‐MOPs*), which exhibit a similar stability profile to their extended MOF counterparts.

Most Zr‐MOPs reported in the literature are based on trinuclear zirconocene nodes and synthesized in situ. This 3‐connected pyramidal cluster was also employed by Yuan et al. to build‐up a series of cationic tetrahedral cages, for which they used 1,4‐benzenedicarboxylic acid (pBDC) and 1,3,5‐benzenetricarboxylic acid (BTC) as organic ligands to obtain the MOPs {[Cp_3_Zr_3_µ_3_‐O(µ_2_‐OH)_3_]_4_[pBDC]_6_}^4+^ (named *ZrT‐1*, with Cp being cyclopentadienyl) and {[Cp_3_Zr_3_µ_3_‐O(µ_2_‐OH)_3_]_4_[BTC]_4_}^4+^ (named *ZrT‐2*), respectively.^[^ ^46^ ^]^ By subjecting the extended carboxylate ligands; 4,4′‐biphenyldicarboxylic acid (BPDC) and1,3,5‐tris(4‐carboxyphenyl)benzene (BTB) to reticular chemistry, the authors obtained two larger MOPs formulated as {[Cp_3_Zr_3_µ_3_‐O(µ_2_‐OH)_3_]_4_(BPDC)_6_}^4+^ (named *ZrT‐3*) and {[Cp_3_Zr_3_µ_3_‐O(µ_2_‐OH)_3_]_4_(BTB)_4_}^4+^ (named *ZrT‐4*), respectively. These ZrT‐1 to ZrT‐4 MOPs showed to be stable in methanol (MeOH) and dimethyl sulfoxide (DMSO). More recently, Su et al. modified pBDC with an amino group to obtain the ligand 2‐aminobenzene‐1,4‐dicarboxylic acid (2‐NH_2_‐pBDC) and then used it to construct a Zr‐MOP of formula {[Cp_3_Zr_3_µ_3_‐O(µ_2_‐OH)_3_]_4_[2‐NH_2_‐pBDC]_6_}^4+^.^[^ ^75^ ^]^ The authors evaluated the stability of this tetrahedral MOP (named *NH_2_‐ZrMOP;* also known as UMOP‐1‐NH_2_) under an aqueous environment from pH= 2 to pH= 10, after which point the NH_2_‐Zr‐cage began to decompose.^[^ ^75^ ^]^ Interestingly, their finding is consistent with the behavior reported for Zr‐based MOFs.^[^ ^72^ ^]^

An alternative method to introduce robust metal nodes into MOP structures entails the use of ligands that form chelates with metal ions, as the chelating effect has been observed to increase the stability of coordination complexes in aqueous media.^[^ ^76^ ^]^ However, the use of ligands with strong coordinating chelating moieties is challenging, as it hinders the necessary reversible bond formation involved in the synthesis of structured metal–organic assemblies such as MOPs. Interestingly, Nitschke and Errington et al. developed an elegant solution: the synthesis of the chelating pyridyl‐imine moiety in situ by reversible covalent chemistry.^[^ ^77^, ^78^ ^]^ However, metal–organic cages based on pyridyl‐imine ligands have been studied mainly in solution; their stability and porous properties in the solid‐state have only recently begun to be investigated.^[^ ^79^, ^80^ ^]^ In 2017, Zhang et al. introduced an alternative, strong‐coordinating, chelate‐based cluster for the synthesis of robust MOPs.^[^ ^81^ ^]^ They reported the synthesis of a tetrahedral Ti(IV)‐based MOP (named *Ti‐MOPs*) assembled from a naphthalene ligand functionalized with adjacent carboxylic and phenol groups. In this Ti‐MOP, three different ligands chelate the Ti(IV) ion, thereby generating a distorted octahedral coordination geometry. Due to the strong chelate‐Ti(IV) coordination, the MOP was stable in water. Alternatively, Tezcan et al. recently introduced the use of hydroxamates as charged chelating groups for the synthesis of Fe(III)‐based MOPs (named *Fe‐MOPs*). Interestingly, they observed a tetrahedral geometry in the Fe(III)‐hydroxamate node, suggesting that this moiety could be used to rationally design other MOPs.^[^ ^82^ ^]^ Given these examples, such existing porous chelate‐based MOPs and others that can be potentially made of highly stable chelates (e.g., metal–catecholates) hold great potential for their future use in subsequent self‐assembly processes.

### 3.3 Solubility

Owing to their discrete molecular structure, MOPs have remarkable solubility in diverse solvents while maintaining their porosity. Accordingly, some MOPs exhibit liquid processability, which can be exploited to perform chemical processes in homogeneous conditions under stoichiometric control. This is highly advantageous for hierarchical assembly of MOPs into porous networks, because self‐assembly can be controlled at the molecular level, thereby avoiding possible sources of anisotropy (e.g., partial reactivity preclusion or aggregation‐induced defects) in the final material. Additionally, the solvent in which the MOP is soluble can strongly influence the self‐assembly process and/or the performance/application of the obtained polymeric network, as observed for hydrogels. Therefore, the solubility profile of the MOP must be understood and controlled before the assembly process. In this regard, MOPs can be readily functionalized—either by direct synthesis or by post‐synthetic modification—with functional groups that enhance their solubility.

The solubilization of MOPs, as for any molecule, is regulated by the balance of the interaction energies of intermolecular attraction in the solid‐state and of solvation in solution. Therefore, the surface of MOPs is critical to solubilization, as it can be used to hinder inter‐MOP interactions (e.g., by using bulky groups to generate steric hindrance) and/or augment MOP‐solvent interactions (e.g., by using hydrophilic or hydrophobic moieties to promote solubility in water or organic solvents, respectively).

Among the first strategies for surface‐functionalizing of MOPs to tune their solubility is the use of pendant functional groups. Interestingly, these organic terminal groups in bridging ligands are largely responsible for the solubility of MOPs. A representative example of this is the cuboctahedral MOP family, based on 5‐functionalized BDC bridging ligands. Yaghi et al. published the first report of a cuboctahedral Cu‐MOP (named *H‐CuMOP*; also known as MOP‐1), having the formula [Cu_2_BDC_2_]_12_, which did not bear any functional group on its surface.^[^ ^36^ ^]^ Therefore, the aggregation of this H‐CuMOP during its synthesis cannot be easily reverted, as solvation is hindered by the strong H‐CuMOP packing interactions, which preclude the solvent molecules and the MOP surface from interacting. Later, the same group reported a surface‐modified isostructural MOP related to H‐CuMOP, whose surface was functionalized with 24 aliphatic dodecyloxy chains by substituting BDC with the 5‐dodecoxybenzene‐1,3‐dicarboxylic acid (5‐C_12_‐BDC).^[^ ^83^ ^]^ This functionalized MOP with formula [Cu_2_(5‐C_12_‐BDC)_2_]_12_ was highly soluble in polar and apolar aprotic organic solvents (e.g., dimethylformamide (DMF) and chloroform, respectively). This approach has been expanded to myriad functional groups to confer the cuboctahedral Cu‐MOP family with a truly broad solubility profile. For example, Zaworotko et al. synthesized hydroxyl‐functionalized cuboctahedral Cu‐MOPs that exhibited high solubility in alcoholic solvents, DMF, and hot acetonitrile.^[^ ^84^ ^]^ Alternatively, Zhou et al. reported that *tert*‐butyl functionalized MOPs were highly soluble in polar solvents such as MeOH and DMF.^[^ ^59^ ^]^ The same authors were the first to introduce charged groups on the surface of cuboctahedral Cu‐MOPs by employing BDC ligands functionalized with sodium sulfonate at the 5‐position (5‐SO_3_‐BDC) to yield a MOP with the formula Na_24_[Cu_2_(5‐SO_3_‐BDC)_2_]_12_ (named SO_3_Na‐CuMOP).^[^ ^59^ ^]^ The exposed sulfonate groups rendered a negatively charged MOP that inhibited MOP aggregation due to electrostatic repulsion. Additionally, they used hydrophilic counter‐cations such as Na(I) to solubilize the SO_3_Na‐CuMOP in water, albeit it hydrolyzed after short incubation times. Conversely, the functionalization of MOPs with strongly hydrophobic groups (e.g., triisopropylsilyl groups) enables their solubilization in highly apolar solvents such as diethyl ether or benzene.^[^ ^85^ ^]^ Examples of ligands used, implementing this effect, include again 5‐functionalized ligands such as 5‐C_12_‐BDC and 5‐hydroxy‐1,3‐dicarboxylic acid (5‐OH‐BDC), which have been combined with highly stable Mo‐ or Rh‐based cuboctahedral cages. A similar strategy has been used in Zr‐MOPs. For example, the solubility of Zr‐based tetrahedral cages in organic solvents such as DMF can be increased by derivatizing them with pendant NH_2_ groups.^[^ ^75^ ^]^ Similarly, Zhou et al. reported a series of geometrically distinct, lantern‐, octahedral‐ and cuboctahedral‐type MOPs based on a quadruple‐bonded Mo(II) paddlewheel combined with diverse ligands of various sizes, bending angles, and organic pendant groups. The authors attributed the solubility of the resultant products to the influence of the pendant groups, observing distinct solubility profiles for isostructural cages that differed only in the functionality of such surface moieties.^[^ ^68^ ^]^

Interestingly, solubility provided by the pendant group in MOPs can be further tailored through covalent post‐synthetic modification. Bloch et al. demonstrated this phenomenon with two cuboctahedral cages, of the formulas M_24_(5‐OH‐BDC)_24_ and M_24_(5‐NH_2_‐BDC) (where M = Cr, Mo, Cu and 5‐NH_2_‐BDC = 5‐amino‐1,3‐benzenedicarboxylic acid), which they functionalized with esters and amides, respectively.^[^ ^86^ ^]^ The authors employed a notably large library of functional groups in their study and, for the amide‐functionalized cages, ascertained the influence of the ligands on the solubility of the resulting cages. They found that introduction of alkane‐ and diphenyl‐groups into the parent MOP enhanced solubility in volatile organic solvents such as dichloromethane, chloroform, tetrahydrofuran, and acetone. Alternatively, our group previously developed a protection/deprotection strategy to synthesize the first‐ever (reported) example of a MOP bearing free carboxylic groups on its surface.^[^ ^54^ ^]^ The synthesized cuboctahedral Rh‐MOP of formula [Rh_2_(BTC)_2_]_12_ (named *COOH‐RhMOP*) was highly soluble in polar solvents such as DMF.

Zhou et al. described a unique mechanism for tuning the solubility of cuboctahedral Cu‐MOPs, based on isomerization of pendant azobenzene groups at the MOP surface.^[^ ^87^ ^]^ First, they linked Cu(II) ions to the ligand 5‐(2,4‐dimethylphenyl)diazinyl)benzene‐1,3‐dicarboxylic acid (L1) to obtain a cuboctahedral Cu‐MOP, with the formula [Cu_2_(L1)_2_]_12_ (named *srMOP‐1*). The azobenzene moieties of the as‐made srMOP‐1, which the authors identified as the *trans*‐isomer, induced strong inter‐MOP interactions through *π*‐*π* interactions, thereby limiting the solubility of *trans*‐srMOP in organic solvents such as MeOH or chloroform. Next, the authors irradiated srMOP‐1 with UV‐light to trigger the *trans‐to‐cis* isomerization of the azobenzene groups, which disrupted the inter‐MOP interactions and consequently, making *cis*‐srMOP‐1 soluble in organic solvents. Their photo‐switching mechanism holds great potential to control the release of active molecules as well as for spatiotemporal control, in situ, of the amount of soluble MOP available for polymerization reactions.

**Figure 3:**
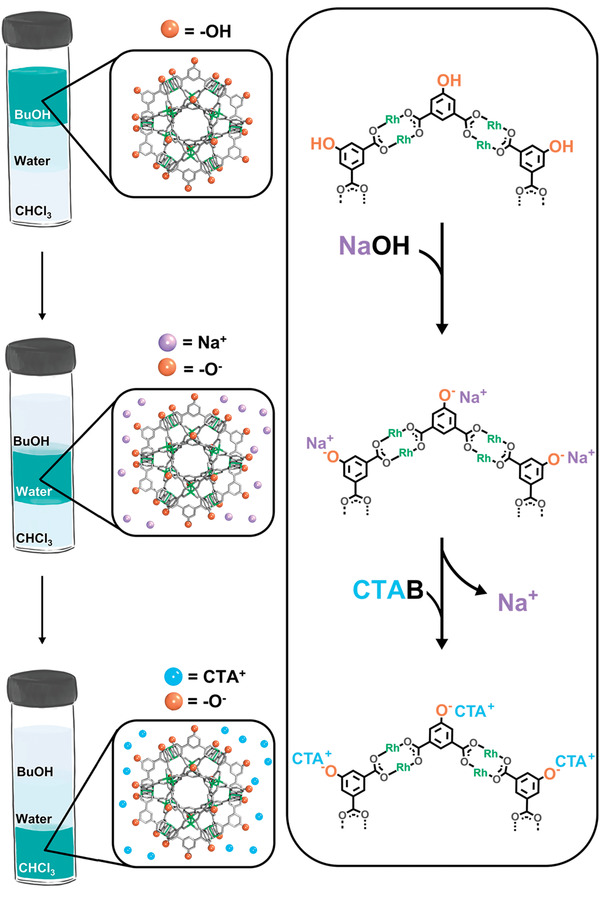
Schematic illustration of the control over solubility dependent on the pendant organic groups.

To date, the role of pendant organic groups on the solubility of MOPs has been investigated according to their intrinsic characteristics or covalent reactivity. However, MOPs bearing pH‐responsive groups can undergo alternative chemical modifications that are based on their protonation state and consequently, can be selectively pH‐triggered. These processes alter the surface chemistry of MOPs and, consequently, affect their solubility. We exploited this concept to tune, in situ, the solubility of a hydroxyl‐functionalized Rh‐MOP of formula [Rh_2_(5‐OH‐BDC)_2_]_12_ (named *OH‐RhMOP*).^[^ ^70^ ^]^ We showed that the 24 hydroxyl groups at the periphery of OH‐RhMOP could be stoichiometrically deprotonated with NaOH to afford a negatively charged MOP of formula Na_24_[Rh_2_(5‐OH‐BDC)_2_]_12_ (named *ONa‐RhMOP*). We observed that ONa‐RhMOP was soluble in aqueous solution, which is consistent with the ability of water molecules to solvate charged species in the presence of hydrophilic counter‐ions. We further demonstrated the crucial role of counter‐cations in dictating the solubility of the charged MOP. We discovered that, analogously to what had previously been reported in inorganic nanoparticles or other metal–organic cages, the sodium ions in ONa‐RhMOP could be substituted with organic cations such as cetyltrimethylammonium (CTA), which afforded a lipophilic MOP of formula CTA_24_[Rh_2_(5‐OH‐BDC)_2_]_12_ that was highly soluble in chloroform (**Figure** **3**).

The aforementioned examples reflect the remarkable role of the pendant groups in defining the solubility profile of MOPs. The polarity, hydrophilicity/hydrophobicity, charge, and counter‐ions of these groups govern the solvation of MOPs by solvent molecules and consequently, their solubilization. Nevertheless, despite the predominant role of these groups in dictating the solubility of MOPs, the metallic nodes also contribute to solubility. For instance, the trinuclear zirconocene cluster found in Zr‐MOPs is inherently positively charged which inhibits strong inter‐MOP interactions, thereby facilitating solubilization of these MOPs in polar solvents.^[^ ^88^ ^]^ Additionally, these metallic cluster undergo cation‐exchange reactions that can be used to further tune solubility. For example, Bloch et al. used ion‐exchange to increase the solubility in methanol of a MOP with the formula Cl_4_{[CpZrµ_3_‐O(µ_2_‐OH)]_3_[Me_2_‐pBDC]_6_} (named *Me_2_‐ZrMOP)* with Me_2_‐BDC being 2,5‐dimethyl‐1,4‐benzenedicarboxylic acid, by exchanging the initial chloride with trifluoromethanesulfonate as counter‐cation.^[^ ^88^ ^]^

An indirect strategy to modify the solubility of MOPs is to leverage the coordination chemistry of their metallic nodes to localize axial ligands toward their periphery, as these ligands ultimately modify the overall surface chemistry of MOPs and consequently, their solubility. Our group, together with the team of Furukawa, have demonstrated the potential of this approach by functionalizing cuboctahedral Rh‐MOPs with N‐donor ligands containing diverse functional groups.^[^ ^38^ ^]^ Specifically, we found that the solubility of [Rh_2_(BDC_2_)]_12_ (named *H‐RhMOP*) and OH‐RhMOP could be modulated by functionalizing their 12 surface di‐rhodium axial sites with distinct N‐donor ligands. We observed that the solubility of the resulting functionalized Rh‐MOP correlated with the hydrophilicity/hydrophobicity of the N‐donor ligand used to functionalize them. For example, L/D‐proline as axial ligand afforded water‐soluble Rh‐MOPs, whereas 4‐(trifluoromethyl)pyridine as axial ligand yielded MOPs soluble in halogenated or aprotic organic solvents such as dichloromethane or tetrahydrofuran. Remarkably, the coordinative solubilizer method applied to Rh‐MOPs does not imply any irreversible structural modification in the cage, as the coordinating ligand can be removed by ligand exchange or by protonating the donor group, in either case without compromising the structural stability of the cages.^[^ ^70^ ^]^ Therefore, this strategy does not prevent the use of functionalized MOPs in subsequent coordination‐driven polymerization reactions (vide infra).

Importantly, the surfaces of some MOPs are functionalized with capping ligands that fulfill the coordination requirements of the MOP's constituent metal ions. Regarding the potential impact of these ligands on the solubility profile of MOPs, we believe that they may have been overlooked in the literature, as indicated by the fact that they have been previously employed to tune the solubility of related non‐porous Pd(II)‐based discrete metal–organic assemblies.^[^ ^89^ ^]^ This strategy may be applicable to permanently porous Zr‐MOPs, which contain on their surface three capping Cp moieties per Zr(IV) cluster. Each of these Cp ligands can be functionalized or exchanged by other capping ligands to tune the surface chemistry and solubility of Zr‐MOPs. Similarly, the solubility of calixarene‐capped MOPs could be modulated by tuning exposed functional group of the macrocyclic capping ligand.^[^ ^90^, ^91^, ^92^, ^93^, ^94^ ^]^

## 4 Assembly of MOPs into Porous Networks

### 4.1 Coordination‐Driven Polymerization

The reactivity of exposed open metal sites in MOPs has been harnessed to drive the assembly of MOPs into crystalline as well as amorphous porous networks. Accordingly, M(II) paddlewheel‐based MOPs, with accessible reactive metal sites, are excellent candidates for coordination‐driven MOP polymerization reactions. This approach benefits from the fact that the equatorial carboxylate‐M(II) coordination bonds of M(II) paddlewheels are more robust than their axial coordination sites. Thus, one can target such reactive sites to polymerize MOPs without compromising their backbone structure. This can be achieved by two different types of coordination reaction: self‐condensation of MOPs functionalized with coordinating moieties on their surface; and linkage of MOPs via ditopic N‐donor ligands. Alternatively, the possibility of functionalizing MOPs with strong coordinating groups (e.g., carboxylic acids) while avoiding MOP self‐condensation has enabled the assembly of MOPs via coordination using bridging metal ions/clusters.^[^ ^42^ ^]^

#### 4.1.1 Self‐Condensation of Functional MOPs

The self‐condensation of MOPs is based on the fact that they can combine open metal sites and coordinating groups from their constituent organic ligands. Zaworotko et al. exploited this feature to assemble MOPs into crystalline networks for the first time.^[^ ^95^ ^]^ They showed that cuboctahedral Cu‐MOPs with reactive surface groups self‐polymerized through sulfonate‐Cu(II) or methoxy‐Cu(II) coordination into 3D or 1D networks, respectively. These networks are distinguished by the fact that their cages are held together chiefly by a strong metal–ligand coordination bond, rather than by the weak, intermolecular, van der Waals interactions typically found in previously reported MOP packing structures. Niu et al. extended this approach by isolating the target MOP before its self‐condensation reaction to achieve a higher degree of control over the final framework structure.^[^ ^55^ ^]^ To this end, they synthesized a cuboctahedral Cu‐MOP of formula [Cu_2_(5‐OH‐BDC)_2_]_12_ (named *OH‐CuMOP*) functionalized with 24 hydroxyl groups. The reactivity of this OH‐CuMOP was modulated by the solvent in which it was solubilized. For most MOPs, solvent molecules tend to coordinate to open metal sites, thereby influencing their surface chemistry. Indeed, bulky solvents such as dimethylacetamide and DMSO imposed steric hindrance around the MOP, which precluded the self‐condensation reaction. However, when the authors dissolved OH‐CuMOP in less‐coordinating and bulky solvents such as methanol, it spontaneously assembled into a 3D structure bridged via Cu(II)‐phenolic hydroxyl coordination of adjacent MOPs. Interestingly, they were able to exploit the presence of small quantities of DMSO in the methanolic reaction mixture to modulate the steric hindrance around the MOPs, partially masking some of its open metal sites and reducing the connectivity of the OH‐CuMOP, thereby enabling synthesis of layered 2D networks.^[^ ^55^ ^]^ This example highlights how previously unconsidered parameters, such as the used solvent, can heavily influence the surface chemistry of MOPs and their self‐assembly reactivity.

An alternative manner to control the assembly of functional MOPs is to temporally occlude their latent reactivity by using protecting groups. For example, Bloch et al. reported that self‐polymerization of a protected NH_2_‐functionalized, lantern‐type Cu‐MOP of formula [Cu_2_(L2)_2_]_12_ (where L2 is 3,3′‐((2,5‐diamino‐1,3‐phenylene)bis(ethyne‐2,1‐diyl))benzoic acid) could be triggered by unmasking reactive groups in solution.^[^ ^96^ ^]^ However, their strategy demanded that the protected MOP withstand the deprotection conditions, which for Cu(II)‐based cages, might limit the scope of compatible protecting groups. Indeed, the authors showed that, although the protected MOP could not withstand the high temperature or strong acids required to remove di‐*tert*‐butyl dicarbonate protecting groups, it was stable to the alkaline conditions required to remove protecting groups such as fluorenylmethyloxycarbonyl. Interestingly, the authors correlated the self‐polymerization rate of protected MOP to the amount of base added to the reaction media, as the latter determined the rate of formation of the reactive groups (i.e., pendant amines). The coordination polymers that they obtained were amorphous, nanoscopic (size: < 50 nm), spherical nanoparticles. However, they found all the coordination polymers to be permanently porous to N_2_ and CO_2_, with great uptake values directly correlating to the polymerization rate. The authors reasoned that at higher self‐polymerization rates, the MOPs pack less efficiently, thereby generating larger pore volumes in the coordination‐polymer products.

#### 4.1.2 Assembly of MOPs through N‐Based Ditopic Ligands

N‐donor ligands exhibit an attractive balance between coordination strength and directionality for M(II) paddlewheel axial sites, thus making pyridine‐, amine‐ and imidazole‐based ditopic ligands suitable for coordination‐driven polymerization of MOPs. The use of these ligands to link MOPs has enabled the scope of MOP‐based networks to widen, as it precludes the standard prerequisite that only inherently reactive MOPs can be used. Additionally, the geometry and the degree of flexibility of N‐based ligands can be harnessed to direct the distance, symmetry, and periodicity between adjacent MOPs, which in turn shape the final assembled material. Thus, this approach has successfully been employed both with rigid ligands, to assemble MOPs into crystalline MOFs, and with flexible ligands, to produce amorphous soft materials.

##### Crystalline MOFs

**Figure 4:**
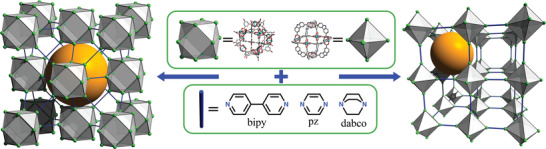
Schematic representation of the assembly of MOPs using rigid N‐ditopic linkers, highlighting the generated interMOP cavity (orange sphere).

Zhou et al. and Chun et al. were the first ones to show the feasibility of crosslinking previously synthesized and isolated MOPs through ditopic N‐based ligands to obtain MOFs.^[^ ^97^, ^98^ ^]^ By applying the supermolecular building block approach to MOPs with well‐defined polyhedral shapes, and rigid or straight ligands, researchers have been able to anticipate the topology of the resultant structures.^[^ ^99^, ^100^ ^]^ For example, the linkage of octahedral Cu‐MOPs of formula [Cu_2_(CDC)_2_]_12_ (where CDC is 9H‐carbazole‐3,6‐dicarboxylic acid) through 4,4′‐bipyridine (bipy), generated a 6‐connected (6‐c) **pcu** network,^[^ ^97^ ^]^ whereas the connection of cuboctahedral Zn(II)‐based MOPs (named *Zn‐MOPs*) of formula [Zn_2_(5‐Me‐BDC)_2_]_12_] (where 5‐Me‐BDC is 5‐methyl‐1,3‐dicarboxylic acid) through 1,4‐diazabicyclo[2.2.2]octane (dabco), resulted in a MOF with an underlying 12‐connected (12‐c) **fcu** topology (**Figure** **4**).^[^ ^98^ ^]^ Later, Su et al. employed the same strategy to tune the structure and composition of MOFs with **fcu** topology, using a Cu‐MOP precursor bearing free amino groups at its surface, of formula [Cu_2_(5‐NH_2_‐BDC)_2_]_12,_ (*NH_2_‐CuMOP;* also known as MOP‐15). Thus, they assembled the NH_2_‐CuMOP with bipyridine to obtain an **fcu** MOF in which the inter‐MOP cavities were derivatized with pendant amino groups.^[^ ^56^ ^]^ The same authors also demonstrated that the inter‐MOP space could be systematically expanded by increasing the length of the pyridine based ligand, from pyrazine to 1,2‐di(pyridin‐4‐yl)ethane.^[^ ^57^ ^]^

Furthermore, given that the polyhedral shape of a MOP precursor determines the final topology of the MOF, the dimensionality of the assembled structure can also be reduced by employing MOPs with fewer vertices or paddlewheel units in their structure. For example, Klosterman et al. used a lantern type Cu‐MOP as precursor to synthesize a 1D coordination polymer.^[^ ^101^ ^]^ The above examples illustrate how strategic choice of MOP and ligand precursors enables the design of dimensionality, framework topology, and/or inter‐MOP chemical space of MOP‐to‐MOF structures.

As previously stated, the solubility of a MOP in a given reaction medium strongly influences the assembly of MOPs into extended structures. However, Lah et al. demonstrated that MOPs could also be used in solid state providing excellent templates for generating hollow MOF structures.^[^ ^102^ ^]^ They implemented this approach by soaking single crystals of OH‐CuMOP in a methanolic solution containing N‐donor rigid ligands (dabco, pyrazole, and bipy). Under these reaction conditions, two processes were coupled: *(i)* solubilization of surface MOP units from the MOP crystal into the methanol phase; and *(ii)* the in situ cross‐linking of the solubilized MOPs with the ligand, which generated a shell of the **pcu** MOF on the MOP crystals. As the reaction progressed, the authors obtained hollow single crystals of the **pcu** MOF. Later, Choe et al. demonstrated that single crystals of the same MOP could be transformed into a **pcu** MOF through a single‐crystal to single‐crystal process comprising immersion of OH‐CuMOP crystals into a DMF/DMSO solution containing dabco.^[^ ^103^ ^]^ The success of this approach relies on the fact that the **fcc** packing structure of the MOP units in the OH‐CuMOP crystal is related to the position of the MOP units in the **fcu** MOF. Thus, slight rotation and translations of the MOP units in the MOP crystal enable accommodation of dabco ligands inside the final MOF structure. Interestingly, by quenching the reaction at intermediate times before the completion of the single‐crystal to single‐crystal transformation, the authors were able to isolate MOP@MOF superstructures. Subsequent selective removal of the MOP core through methanol washings enabled synthesis of single crystalline MOF hollow structures. Further MOP growth on the core shell structures, followed by MOF synthesis and subsequent MOP removal, enabled synthesis of matryoshka and double‐shell hollow structures.

##### Soft Porous Materials

By assembling MOPs into amorphous soft materials, researchers can design porosity into materials that are typically non‐porous, such as amorphous coordination polymers. Compared to crystalline networks, these materials have the advantage of a higher degree of processability, as their porosity does not rely on long‐range order. Interestingly, MOPs have been polymerized into soft porous materials through use of short N‐based flexible ligands. Chun et al. were the first to employ this approach, in which they polymerized Cu‐MOPs into amorphous coordination polymers using flexible diamine ligands, such as ethylenediamine, xylylenediamine, and diaminoheptane.^[^ ^104^ ^]^ Crucially, all assembled coordination polymers exhibited permanent porosity, as assessed by N_2_, H_2,_ and CO_2_ adsorption measurements at low temperature. Furthermore, the amorphous coordination polymers displayed bimodal porosity: the authors ascribed contributions from the microporous regime to the inner MOP cavity, and from the mesoporous regime to the inter‐MOP voids. Following the flexible ditopic ligand approach, Furukawa et al. synthesized intrinsically porous amorphous coordination polymers by assembling C_12_ *‐*RhMOP with the imidazole‐based ligand 1,4‐bis(imidazole‐1‐ylmethyl)benzene (bix).^[^ ^105^ ^]^ This assembly process was dictated by the quantity of bix ligand added to the C_12_‐RhMOP solution. The stepwise addition of bix to the C_12_‐RhMOP solution triggered a polymerization reaction that followed a nucleation‐elongation mechanism to yield spherical nanoparticles. Alternatively, addition of C_12_‐RhMOP to a solution containing excess bix (12 molar eq. relative to MOP) in a single portion, resulted in formation of a kinetically trapped molecule in which all the Rh(II) axial sites of the C_12_‐RhMOP were occupied by bix ligands monodentate coordination, to give a MOP of formula [Rh_2_(5‐C_12_‐BDC)_2_(bix)]_12_ (named *C_12_‐RhMOP(bix)_12_*). Self‐assembly of such C_12_‐RhMOP(bix)_12_ in solution was triggered by heating the solution at 80 °C. Under these conditions, a fraction of the monodentate bix was detached from the surface of the MOP, generating vacancies that enabled crosslinking of the MOPs via formation of bidentate bix bridges. This in turn generated a colloidal suspension that, upon further incubation at 80 °C, assembled into a colloidal gel in situ. In terms of functional porosity, both forms of the coordination polymer (colloidal particles and supramolecular gels) outperformed the initial C_12_‐RhMOP, as assessed by N_2_ and CO_2_ sorption measured at 77 and 195 K, respectively. Specifically, N_2_ adsorption measurements displayed a negligible adsorption of 0.17 mol(N_2_)/mol(C_12_‐RhMOP) for the discrete C_12_‐RhMOP, and uptakes of 8.70, and 18.61 mol(N_2_)/mol(C_12_‐RhMOP) for the colloidal particles and supramolecular gel, respectively.^[^ ^105^ ^]^

Furukawa et al. studied the gelation of kinetically trapped porous building blocks into colloidal gels by time‐resolved dynamic light scattering, for both kinetic analysis of the gelation process itself as well structural characterization of the colloidal network.^[^ ^39^ ^]^ The structural data were important, as the structure of these gels determines their viscoelastic properties. In this sense, the authors discovered that the gel network depends mainly on the concentration of the initial C_12_‐RhMOP(bix)_12_ precursor. Additionally, through mechanistic analysis, the authors determined that the main parameters governing the gel network structure (density, correlation length, and degree of branching) are established at the stage in which the colloids aggregate through attractive coordinating forces, just before percolation. Based on this discovery, the authors devised a novel methodology to selectively confer gels with a continuous and gradual change in mechanical properties. Their strategy is based on using centrifugal force to create a gradient of pre‐gelating, reactive colloidal particles, which are then incubated at high temperature to induce gelation. Since the concentration of reactive colloidal gels varies across the height of the gel, so do the mechanical properties of each final gel. More recently, Furukawa et al. also showed an alternative way to trigger the gelation of C_12_‐RhMOP(bix)_12_ via acid‐triggered detachment of bix.^[^ ^40^ ^]^ They found that addition of stoichiometric amounts of trifluoroacetic acid (TFA) to a DMF solution of C_12_‐RhMOP(bix)_12_ triggered gelation at room temperature. By replacing TFA with the photoacid 8‐hydroxypyren‐1,3,6‐trisulfonic acid (pyranine), they were able to synthesize porous gels upon light irradiation at room temperature, thereby enabling photo‐patterning of the gel.

**Figure 5:**
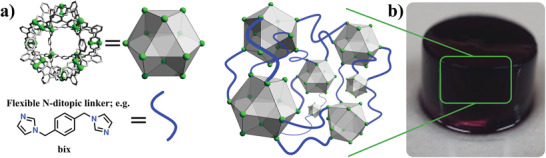
a) Schematic illustration of the assembly of MOPs as porous monomers through flexible ditopic linkers to form amorphous supramolecular polymers with intrinsic porosity arising from the MOP units. b) Photograph of a supramolecular colloidal gel resulting from this assembly. Reproduced with permission.^[^^]^ Copyright 2019, Wiley‐VCH.

The long aliphatic chains on the surface of the C_12_‐RhMOP used to synthesize MOP‐based gels ensure the solubility of the MOP precursor. However, these chains are detrimental to the porosity of the derived aerogels, as they occupy free volume in the final assembly, thus hindering gas diffusion. Nevertheless, as explained above, MOPs can also become soluble through reactivity of their axial site. Accordingly, our group, together with Furukawa et al., developed a methodology to assemble the highly porous but insoluble H‐RhMOP into colloidal particles and gels (**Figure** **5**b).^[^ ^106^ ^]^ First, the long aliphatic imidazole, 1‐dodecyl‐1H‐imidazole, was attached to the open metal sites of H‐RhMOP to make it soluble in organic solvents. Next, bix was added to the functionalized H‐RhMOP, which triggered its self‐assembly into colloidal particles via ligand exchange reaction. Remarkably, this strategy enabled us to increase the porosity of the derived aerogels, which showed bimodal porosity and BET surface areas (S_BET_) of up to 540 m^2^ g^–1^. Moreover, Furukawa et al. were able to augment the porosity of the H‐RhMOP‐derived gels through a post‐synthetic gel maturation strategy.^[^ ^107^ ^]^ They found that, upon incubation at 80 °C, the colloidal gel network became more porous due to greater inter‐MOP crosslinking, reaching an S_BET_ value of 758 m^2^ g^–1^. More importantly, this work highlights how molecular scale phenomena (e.g., MOP crosslinking) span multiple length scales up to the macroscale (e.g., gel densification) to influence the final properties (e.g., gas adsorption) of functional products. Alternatively, MOP‐based hydrogels can be prepared by employing the counteraction solubilization approach. For example, Furukawa et al. reacted the water‐soluble ONa‐RhMOP with bix in a mixture of acetonitrile and water to obtain a colloidal gel.^[^ ^108^ ^]^ Subsequent water exchange treatment afforded a hydrogel with no traces of organic solvent.

#### 4.1.3 Assembly of MOPs through Coordination with Additional Metal Ions

**Figure 6:**
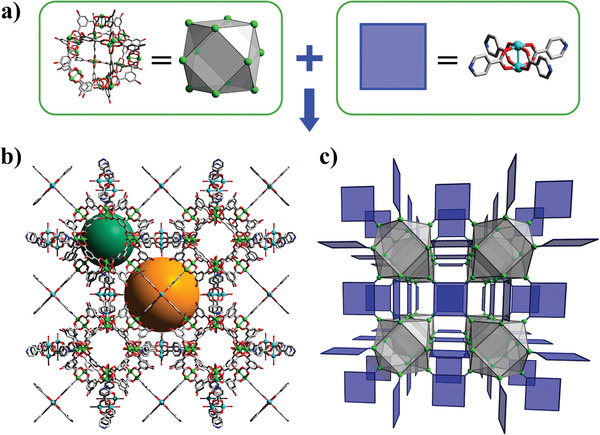
a) Fragments of the structure of RhCu‐**ftw**‐MOF, highlighting the connectivity of OH‐RhMOP(HINA)_12_ (12‐c SBB) through the 4‐c Cu_2_ paddle‐wheel unit. b) Structure of RhCu‐**ftw**‐MOF, highlighting the generated interMOP cavity (orange sphere). c) Illustration of the **ftw** topology.

Recently, our group introduced a novel methodology that differs from the approaches that we have discussed so far, such as self‐condensation of MOPs, or linking of MOPs via rigid ditopic ligands. Our approach consists of introducing pendant and available carboxylic acid groups onto the periphery of a cuboctahedral Rh‐MOP, and then harnessing the reactivity of these groups to assemble COOH‐RhMOPs with additional metal ions.^[^ ^42^ ^]^ The chemistry of Rh‐MOPs enabled us to precisely position carboxylic acid groups on the 24 edges or the 12 vertices of their cuboctahedral surfaces, through a covalent or coordination post‐synthetic route, respectively. The Rh(II) axial sites of the OH‐RhMOP were used to anchor 12 isonicotinic acid (HINA) molecules onto its vertices via Rh‐pyridine coordination to obtain a MOP with formula [Rh_2_(5‐OH‐BDC)_2_(HINA)]_12_ (named *OH‐RhMOP(HINA)_12_*), unaltering the carboxylic acids. Topologically, the resulting OH‐RhMOP(HINA)_12_ can be described as a 12‐c cuboctahedral supermolecular building block. We prepared the analogous 24‐connected (24‐c) rhombicuboctahedral supermolecular building block COOH‐RhMOP, following our own protocol, entailing use of stoichiometric protecting groups. Both COOH‐functionalized MOPs were soluble in organic solvents and, owing to the low chemical affinity of carboxylic acid groups for the Rh(II) axial sites, did not undergo any self‐condensation. However, addition of Cu(II) ions to OH‐RhMOP(HINA)_12_ and COOH‐RhMOP triggered their self‐assembly into crystalline networks of the topologies (4,12)‐c **ftw** and (3,24)‐c **rht**, respectively. The final accessed topology for each of the COOH‐functionalized MOPs agreed with the outcome that we had expected upon considering the connectivity and geometry of each of the COOH‐RhMOPs. Interestingly, Cu(II) adopts an uncommon coordination environment, forming a Cu(II) trimer in the **rht** structure to satisfy the steric and connectivity requirements of the (24‐c) COOH‐RhMOP. From a chemical perspective, the assembly of up to 4 different molecular components into a single compositionally complex structure, such as in the **ftw** network, is enabled by a clear gradient in coordination strength of the different coordination bonds present in the multi‐component structure (**Figure** **6**). This hierarchy in coordination strength is what prevents the Cu(II) ions from replacing the Rh(II) ions in the MOP structure, and prevents the HINA from detaching from the MOP in the presence of Cu(II). Therefore, the stepwise assembly of MOPs functionalized with well‐defined points of extension through hierarchic or orthogonal interactions provides the opportunity to construct multi‐component MOF structures with a predefined topology and sequence of its different constituent building blocks.

### 4.2 Assembly of MOPs through Supramolecular Non‐Coordinative Bonds

**Figure 7:**
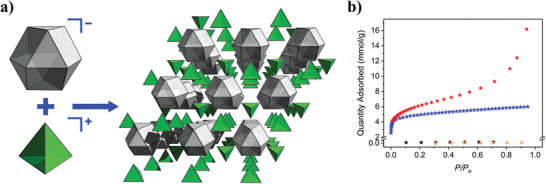
a) A portion of the crystal structure of the doubly porous salt X_16_[Me_2_‐ZrMOP]_2_[SO_3_‐CuMOP]. Grey and green polygons represent the cuboctahedral SO_3_‐CuMOP and the tetrahedral Me_2_‐ZrMOP, respectively. b) N_2_ adsorption isotherm at 77 K of the porous salt (red), Me_2_‐ZrMOP (blue), and SO_3_‐CuMOP (black). Reproduced with permission.^[^ ^88^ ^]^ Copyright 2020, American Chemical Society.

The supramolecular reactivity of MOPs is not limited to coordination chemistry: their surfaces can be functionalized with organic moieties able to establish alternative non‐covalent interactions such as *π*‐*π* stacking, H‐bonding, and electrostatic or hydrophobic/hydrophilic interactions. Indeed, such interactions are commonly observed in as‐made MOP packing structures.^[^ ^109^ ^]^ However, the deliberate use of non‐coordinating supramolecular interactions for the hierarchical construction of MOP‐based networks is currently in its infancy. Bloch et al. recently provided the first example of electrostatically driven assembly of MOPs.^[^ ^88^ ^]^ The authors used two oppositely charged MOPs as building blocks to synthesize the first intrinsically porous salt. Reaction of the positively charged Me_2_‐ZrMOP with a negatively charged Cu‐MOP of formula [Cu_24_(5‐SO_3_‐BDC)_12_]^24−^ (named *SO_3_‐CuMOP*) gave rise to amorphous powders, in which the charge is balanced exclusively by the presence of charged MOPs (i.e., 6 Me_2_‐ZrMOPs for 1 SO_3_‐CuMOP). The amorphous porous salt exhibited greater porosity than its building blocks, due to the removal of non‐porous counter‐ions from the original structure, displaying a S_BET_ of 496 m^2^ g^–1^ for the amorphous porous salt, against the S_BET_ of 416 m^2^ g^–1^ and non‐porous of the individual Me_2_‐ZrMOP and SO_3_‐CuMOP, respectively (**Figure** **7**b). Interestingly, the presence of large counter‐ions in each of the charged precursor MOPs, such as tetraethylammonium for the anionic SO_3_‐CuMOP or triflate for the cationic Me_2_‐ZrMOP, enabled the authors to isolate a crystalline salt of formula X_16_[Me_2_‐ZrMOP]_2_[SO_3_‐CuMOP] (where X is H^+^ or tetraethylammonium cations) (Figure 7). The authors suggested that the use of larger counter‐ions likely slowed down the metathesis reaction, enabling formation of the crystalline product. Later, they expanded their approach to create a large library of novel porous salts, by combining an array of positively charged and negatively charged MOPs.^[^ ^110^ ^]^

Interestingly, Ohba et al. used a similar strategy to demonstrate that bulky counter‐ions could be used to control assembly of charged MOPs.^[^ ^111^ ^]^ They assembled Me_2_‐ZrMOP with the polyoxometalates (POMs) of the formula [SiW_12_O_40_]^4−^. Consistent with the charge of each building block, the resultant POM‐MOP crystalline assembly has a MOP/POM ratio of 1:1. Furthermore, the POM‐MOP network is permanently porous in the solid state exhibiting an S_BET_ up to 425 m^2^ g^–1^, making the POM units highly accessible within the network.

### 4.3 Covalent Polymerization

**Figure 8:**
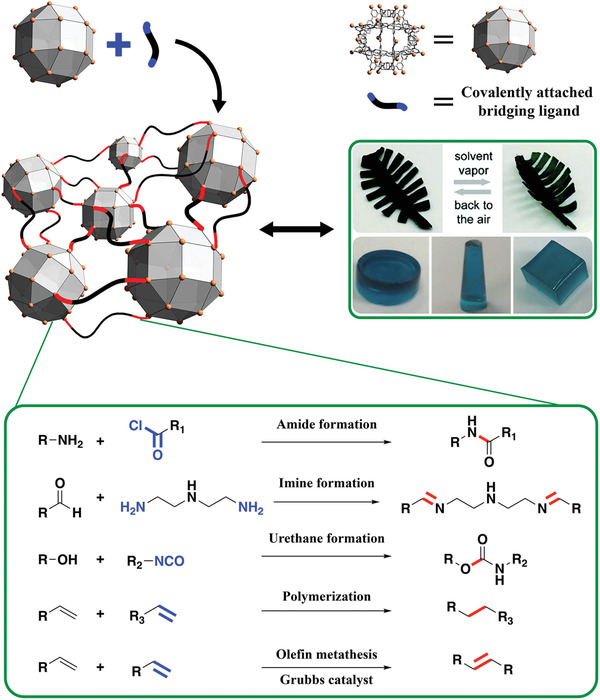
Schematic representation of the covalent polymerization and the corresponding cross‐linking reactions. Highlighted in red are the newly formed covalent bonds and in blue the participating functionality on the bridging ligand. Photos of green leaves: Reproduced with permission.^[^ ^116^ ^]^ Copyright 2020, Royal Society of Chemistry. Phtos of blue gels: Reproduced with permission.^[^ ^119^ ^]^ Copyright 2019, Royal Society of Chemistry.

Covalent bonds are amongst the strongest chemical bonds. Therefore, their use to link MOPs holds great potential to develop robust MOP‐based networks. Additionally, the mature field of polymer science can be used as a guide to develop synthetic methodologies that enable shaping of MOP‐based networks into diverse functional macroscopic materials such as gels, monoliths, or thin films. However, there are few literature reports on using covalent bonds to assemble MOPs into porous networks, underlining the difficulty of this approach. Possible drawbacks include the stability and/or solubility of the MOPs (vide supra) under the required coupling conditions (i.e., organic solvents at high temperatures in the presence of potentially coordinating reagents). Interestingly, most of the literature examples are condensation (formation of amides, urethanes, or imines)^[^ ^112^, ^113^, ^114^, ^115^, ^116^ ^]^ or olefin‐based cross‐linking (polymerization or metathesis)^[^ ^117^, ^118^, ^119^, ^120^, ^121^ ^]^ (**Figure** **8**).

#### 4.3.1 Cross‐Linking Based on Condensation Processes

The first example of cross‐linking MOPs through covalent chemistry was provided by Choe et al. in 2017.^[^ ^112^ ^]^ The authors reacted NH_2_‐ZrMOP with acyl chloride ligands (6 to 10‐membered carbon chains) to cross‐link the MOPs through amide bonds.^[^ ^112^ ^]^ They ran the reaction under heterogeneous conditions, with the NH_2_‐ZrMOP precursor in its solid crystalline state. Interestingly, the crystalline packing and the intrinsic microporosity of the MOPs were retained after condensation. Therefore, this pioneering example illustrates that Zr‐MOPs are sufficiently robust to withstand amide‐bond formation, even when this entails in situ release of HCl. However, the heterogeneous reaction conditions demanded long reaction times (up to 3 days) and offered only limited control over the processability and the macroscopic form of the final MOP network. Intriguingly, to gain greater control over amide‐bond formation and to reduce diffusion pathways, Guo et al. used a water‐soluble NH_2_‐ZrMOP as precursor.^[^ ^113^ ^]^ They reacted NH_2_‐ZrMOP with trimesoyl chloride through an interfacial polymerization method to produce Zr‐MOP‐based thin films. The resultant films exhibited excellent water permeability, dye rejection, and strong antibacterial activity, making them suitable for nanofiltration. Similarly, Zhang et al. fabricated MOP‐based membranes by condensing NH_2_‐ZrMOP with aldehydes to form polyimine networks.^[^ ^114^ ^]^ They ran the reaction under homogeneous conditions, in a mixture of organic solvents, and in the presence of the triamine tris(2‐aminoethyl)‐amine and terephthalaldehyde as co‐monomers to form the hyper‐cross‐linked networks. The membranes were easily obtained upon solvent removal and exhibited high permeability and anionic dye rejection. The membranes also had enhanced mechanical properties, stemming from the highly cross‐linked nature of their network. Alternatively, Volkmer et al. showed that Cu‐MOPs functionalized with hydroxyl groups could be grafted with polymer chains through the formation of urethane bonds.^[^ ^115^ ^]^ Likewise, Yan et al. used this chemical approach to crosslink cuboctahedral Rh‐MOPs into polyurethane networks.^[^ ^116^ ^]^ They synthesized urethane‐reactive MOPs with the formula [Rh_2_(BDC)_m_(5HOC_4_‐BDC)_n_]_12_ (named *HOC_4_‐RhMOP*) by introducing 5‐(4‐hydroxybutoxy)‐1,3‐benzenedicarboxylic acid (5‐HOC_4_‐BDC) into the synthesis of H‐RhMOP with different ratios (*m* = 16, *n* = 8; *m* = 8, *n* = 16 and *m* = 0, *n* = 24). They found that the ratio between the reactive ligand and BDC dictated the reactivity of HOC_4_‐RhMOP. In all cases, upon reacting HOC_4_‐RhMOP with a mixture containing isophorone diisocyanate, polytetramethylene glycol, and 1,4‐butanediol, they obtained elastic MOP‐polymeric materials, which they named ElastoMOPs. Interestingly, they correlated the mechanical properties of the ElastoMOPs (MOP content: 6.5% to 7.8% w/w) to the type of MOP precursor used: those ElastoMOPs with a higher percentage of hydroxyl groups at their surfaces exhibited better mechanical properties, due to their greater degree of cross‐linking. Finally, inspired by nature, the authors used Elasto‐MOP to build a leaf that exhibited biometric movement upon exposure to vapor (see Figure 7, right).

#### 4.3.2 Cross‐Linking Based on Olefins

There are various examples of MOPs undergoing olefin‐based cross‐linking chemistry. For instance, Shimizu et al. cross‐linked a cuboctahedral Cu‐MOP that was partially functionalized with alkyl chains containing terminal alkene groups [5‐(dec‐9‐en‐1‐yloxy)1,3‐benzenedicarboxylic acid (C = C_9_‐BDC) and 5‐(octyloxy)1,3‐benzenedicarboxylic acid (C_18_‐BDC)], through olefin metathesis using a Grubbs catalyst.^[^ ^117^ ^]^ The degree of crosslinking between the alkene‐functionalized MOPs with the ideal formula [Cu_2_(C = C_9_‐BDC)(C_18_‐BDC)]_12_ (named *C = C‐CuMOPs*) could be controlled by the amount of catalyst used. The physicochemical properties of the obtained metathesized networks related to their cross‐linking degree, which varied from 20% to 80% (depending on the catalyst loading). Thus, networks with a higher degree of cross‐linking were insoluble in organic solvents and had superior mechanical properties (i.e., greater hardness and lower elastic modulus). Additionally, the MOP‐networks showed greater adsorption than did the isolated C = C‐CuMOPs, most likely due to the appearance of additional voids at the inter‐MOP space. Another compelling coupling method using olefins to connect MOPs is radical polymerization, as realized by Sun et al.^[^ ^118^, ^119^ ^]^ They derivatized the NH_2_‐CuMOP with methacrylate to make it reactive toward butyl methacrylate, using UV‐light to yield a cross‐linked membrane with a MOP content of up to 10%. The MOP within the membrane was much more stable against hydrolysis than the initial MOP precursor, due to the polymeric shielding effect (vide supra), which enabled the use of these membranes as adsorbents of organic dyes in aqueous media.^[^ ^118^ ^]^ The same authors used an alkene‐functionalized MOP of formula [Cu_2_(5‐(3‐butene‐1‐yloxy)BDC_2_]_12_ (named *Bt‐CuMOP*) as monomer in self‐polymerizing reactions, and as co‐monomer in polymerization reactions with polystyrene.^[^ ^119^ ^]^ In the latter case, the authors used azobisisobutyronitrile and heat as radical initiators, which led to a final composite exhibiting a MOP content of 88% (1 MOP polymerized with 10 styrene molecules). Both strategies yielded covalently linked MOP‐networks with greater hydrolytic stability and better sorption properties relative to the initial Bt‐CuMOP. Additionally, the polystyrene co‐polymerization strategy enabled the synthesis of easy‐to‐shape gels, thus enhancing the processability of the MOP‐networks. Similarly, Zhao et al. subjected an amine‐functionalized Zr‐MOP to post‐synthetic, UV‐light mediated cross‐linking.^[^ ^120^ ^]^ The authors reacted single crystals of NH_2_‐ZrMOP with acryloyl chloride to obtain an acrylated NH_2_‐ZrMOP (named *AA‐ZrMOP*),^[^ ^121^ ^]^ which they then reacted, as soluble precursor, with acrylate‐terminal polyethylene oxide precursors. This cross‐linking reaction gave rise to self‐standing thin membranes with a MOP content of up to 3%. The use of the AA‐ZrMOP as cross‐linking agent in the polymerization reaction later enabled synthesis of membranes with great thermal stability and CO_2_ permeability. The above examples demonstrate that covalent linking can be used to enhance the robustness of synthesized superstructures and thus is ripe for further investigation.

## 5 Perspectives

The longstanding potential of functional MOPs as building blocks for the design and assembly of porous materials is finally beginning to bear fruits, as reflected in an increasing number of reports of their use in hydrogels,^[^ ^122^ ^]^ hybrid composites,^[^ ^123^ ^]^ salts,^[^ ^89^ ^]^ hairy dendrimers,^[^ ^124^ ^]^ and MOFs.^[^ ^42^ ^]^ Indeed, beyond their porosity, MOPs boast an impressive array of other exploitable structural properties, including tunable solubility, high connectivity, and well‐defined peripheral points of extension.^[^ ^71^, ^106^, ^125^ ^]^ However, their practical usage has remained limited due to a lack of robust functionalized MOPs and a poor compatibility with extension chemistry. Fortunately, the development of robust MOPs, either through direct synthesis or post‐synthetic modification, has enabled researchers to harness the surface chemistry of MOPs to modulate their solubility and to open up alternative extension pathways that, whilst demanding more aggressive conditions than in other methods, nevertheless afford materials with a higher degree of specialization and structural integrity.^[^ ^108^, ^112^, ^126^ ^]^ However, there is ample room for improvement in the design and synthesis of stable functional MOPs and their subsequent incorporation into extended materials. Herein, we provide the lector with our insight into the future of the field and highlight the most promising pathways for the synthesis of previously inaccessible materials with cutting‐edge properties.

The first pathway, and a rather straightforward one, is to invest more in developing new MOP cages with directional functionalities poised for extension chemistry,^[^ ^54^, ^96^ ^]^ whether by direct synthesis or post‐synthetic modification. As we previously mentioned, the design of functional MOPs with reactive surfaces had long been hampered by stability issues, which have recently been overcome with the advent new synthetic and post‐synthetic methodologies. To date, researchers have not had any incentive to add new functionalities to MOPs per se; however, there is now a vast landscape of potential pathways for the preparation of extended MOP‐based materials. The importance of further developing this pathway is illustrated by the recent creation of carboxylate‐tagged MOP cages, in which incorporation of such a simple, yet challenging, moiety at the periphery of a Rh‐based cage has laid the first stone toward the formation of crystalline, hierarchically porous, MOF architectures with unusual topologies and well‐defined hybrid metal sites.^[^ ^42^ ^]^ Indeed, although there is only one reported example of this use of carboxylates, it demonstrates the potential for a single functional group to massively expand the potential catalog of synthetically feasible MOPs: thus, thousands of previously inaccessible hierarchical MOF structures become available through the assembly of COOH‐tagged preformed cages, without the need for any uncontrollable metal–ligand exchange reactions or any chemical‐etching.^[^ ^30^, ^127^ ^]^ We anticipate the same effects for other, equally relevant functional groups (acrylates, azide‐alkyne, sequential nucleotides, etc.), once they are used in a similar way on MOP surfaces, whether individually or collectively, to generate novel soft matter and crystalline extended architectures. Thus, there is a need to re‐explore the chemistry behind the basic cage design bearing new horizons in mind. Furthermore, most MOP‐extension chemistry, whether reported or simply postulated, is based on building a single type of cage for simplicity. However, as clearly exemplified in the field of multicomponent MOFs,^[^ ^128^ ^]^ there is a latent possibility of building complex architectures from multiple molecular building blocks, whether nodes or ligands.^[^ ^129^, ^130^ ^]^ Therefore, the same principles could be applied to assemble multiple MOP cages, with distinct geometries and functionality, to obtain extended elaborated architectures through one or more assembly approaches, including H‐bonds, dynamic covalent bonds, or metal‐mediated coordination. Pioneering work on the co‐crystallization of varied MOPs reflects the potential of this approach toward fine tuning of the physicochemical properties of MOP‐based materials.^[^ ^125^, ^131^ ^]^

Another topic that we consider promising for the future is the molecular nature of MOP surfaces, which can enable stoichiometric control over the total number of appended functionalities, such that the final degree of cross‐linking among the cages could be controlled. Indeed, although in this review we have focused on periodically extended materials, the molecular nature of MOPs offers vast potential to explore assembly of finite superstructures through stoichiometric, limited‐growth steps.^[^ ^132^, ^133^ ^]^ At the intersection of molecular materials and periodic materials lies an alluring world of finite architectures (e.g., dendrimers, stepwise polymers, and molecular superstructures) with exceptional properties.^[^ ^134^, ^135^ ^]^ To the best of our knowledge, there is scarcely any representation of coordination‐based porous materials of those types.^[^ ^135^ ^]^ MOPs, with their tunable solubility and finite functionalities, can operate in the same molecular regime as these particular macromolecules. Thus, if researchers could further optimize the stepwise reactivity of the orthogonal surfaces of MOPs and achieve stoichiometric control over the total degree of extension, then they could potentially combine some exciting porous macromolecular materials with unprecedented degrees of control.

Finally, we believe that the internal functionality of MOPs has been underexploited for developing unique extended materials. Unlike other archetypical porous frameworks, those assembled from functionalized MOPs are built from supermolecular building blocks with accessible cavities positioned for host‐guest chemistry, and thus, can be used to periodically space important substrates within the solid‐state. Although the host‐guest chemistry of MOPs is presently not as developed as this approach demands, the same principles could be extrapolated from the more mature host‐guest chemistry of coordination cages. Indeed, coordination cages have rich pathways for encapsulation of target‐solvated guests within their cavities.^[^ ^135^, ^136^, ^137^ ^]^ In fact, such encapsulation stabilizes the guest molecules and can even enhance their reactivity.^[^ ^138^ ^]^ Thus, once researchers extend this concept to MOPs, they will be able to arrange critical substrates within a periodic solid‐state lattice, thereby exposing them to heterogeneous conditions, including solid‐gas phase processes, strongly demanded by industry.^[^ ^139^ ^]^ This approach could be further enriched by ordered combination of different MOPs into porous networks to pave the way for the spatial organization of varied functional guests.

In summary, the use of MOPs in bottom‐up synthesis of porous materials, mostly overlooked, is now a thriving topic in materials science. Recent advances in MOP surface chemistry, and the development of soluble MOP platforms that can withstand aggressive thermal and chemical conditions, together have led to a new generation of highly pre‐organized molecular building blocks with exciting structural properties. This progress is influencing a diverse array of materials, from soft matter to crystalline frameworks, for which researchers are achieving unparalleled levels of structural control thanks to their intrinsic cavities, open metal sites, and orthogonally reactive surfaces. Our review represents merely the tip of the iceberg of the potential of MOPs to contribute to nearly any self‐assembled material, whether discrete or extended, which is built from well‐defined, specialized, molecular building blocks.

## Conflict of Interest

The authors declare no conflict of interest.
